# A real-world single-center study on the effects of low-volume polyethylene glycol electrolyte solution and rhubarb for colonoscopy preparation: quality, tolerance, and experience

**DOI:** 10.3389/fmed.2026.1922646

**Published:** 2026-10-21

**Authors:** Caiwen Tan

**Affiliations:** Department of Gastroenterology, People's Hospital of Kaizhou District, Chongqing, China

**Keywords:** bowel preparation, colonoscopy, gastrointestinal tolerance, polyethylene glycol electrolyte solution, rhubarb

## Abstract

**Background:**

Low-volume polyethylene glycol electrolyte solution (PEG-ELS) combined with adjunctive laxatives may reduce oral fluid burden without compromising bowel cleansing quality. Evidence regarding rhubarb combined with low-volume PEG-ELS remains limited.

**Methods:**

This retrospective single-center cohort included 257 patients undergoing elective colonoscopy from June 2023 to June 2024. Patients received low-volume PEG-ELS plus rhubarb (*n* = 131) or conventional 3-L PEG-ELS (*n* = 126). Primary outcomes were adequate bowel preparation rate and total Boston Bowel Preparation Scale (BBPS) score. Secondary outcomes included defoaming score, bubble-clearance time, insertion and withdrawal times, defecation timing, lesion detection, gastrointestinal adverse reactions, patient experience, and safety. Multivariable analyses adjusted for age, sex, body mass index, and constipation history. Propensity score matching (PSM), inverse probability of treatment weighting (IPTW), and an exploratory constipation subgroup analysis were performed. Prespecified non-inferiority margins were −10% for adequate preparation rate and −1 point for total BBPS score.

**Results:**

Adequate preparation occurred in 94.66% (124/131) of the combination group and 89.68% (113/126) of controls, with a difference of 4.98% (95%CI: −1.54% to 11.50%), meeting the prespecified non-inferiority criterion. Total BBPS score also met the non-inferiority criterion (adjusted *β* = 0.17, 95%CI: −0.05 to 0.40, *P* = 0.136). The combination group had a lower defoaming score (adjusted *β* = −0.20, *P* = 0.020) and shorter bubble-clearance time (18.72 ± 7.64 vs. 27.35 ± 9.12 s, *P* < 0.001), whereas insertion time was similar (6.84 ± 2.16 vs. 7.13 ± 2.38 min, *P* = 0.304). Time to first defecation and total bowel evacuation were shorter, and overall gastrointestinal adverse reactions were less frequent (all *P* < 0.001). Satisfaction and willingness to reuse the regimen were higher (both *P* = 0.010). Lesion detection rates did not differ. PSM and IPTW confirmed the main findings. Among 39 patients with constipation, defecation-related times were shorter with the combination regimen, whereas BBPS, preparation adequacy, defoaming score, and insertion time were similar. No serious bowel preparation-related adverse events were documented.

**Conclusions:**

Low-volume PEG-ELS plus rhubarb maintained bowel cleansing quality while improving defoaming, evacuation efficiency, gastrointestinal tolerance, and patient experience. Improved defoaming did not significantly shorten insertion time. Findings in patients with constipation require prospective randomized confirmation.

## 1 Introduction

Colonoscopy serves as a core tool for colorectal cancer screening, precancerous lesion identification and diagnosis and treatment of colorectal diseases, and its diagnostic quality largely depends on bowel preparation efficacy. Inadequate bowel preparation results in poor mucosal visualization, difficult cecal intubation, prolonged procedural time, potentially reduced detection rates of early lesions, and increased repeated examination and medical resource consumption. Updated consensus recommendations from the US Multi-Society Task Force on Colorectal Cancer emphasize that bowel preparation quality should be regarded as a key component of colonoscopy quality control, and propose strategies including split-dose administration, optimized medication timing, enhanced patient education and individualized regimen selection to elevate adequate bowel preparation rates (1). Therefore, an ideal bowel preparation regimen should guarantee sufficient cleansing while balancing safety, compliance, gastrointestinal tolerance and patients' willingness to undergo repeated examinations.

**Figure 1:**
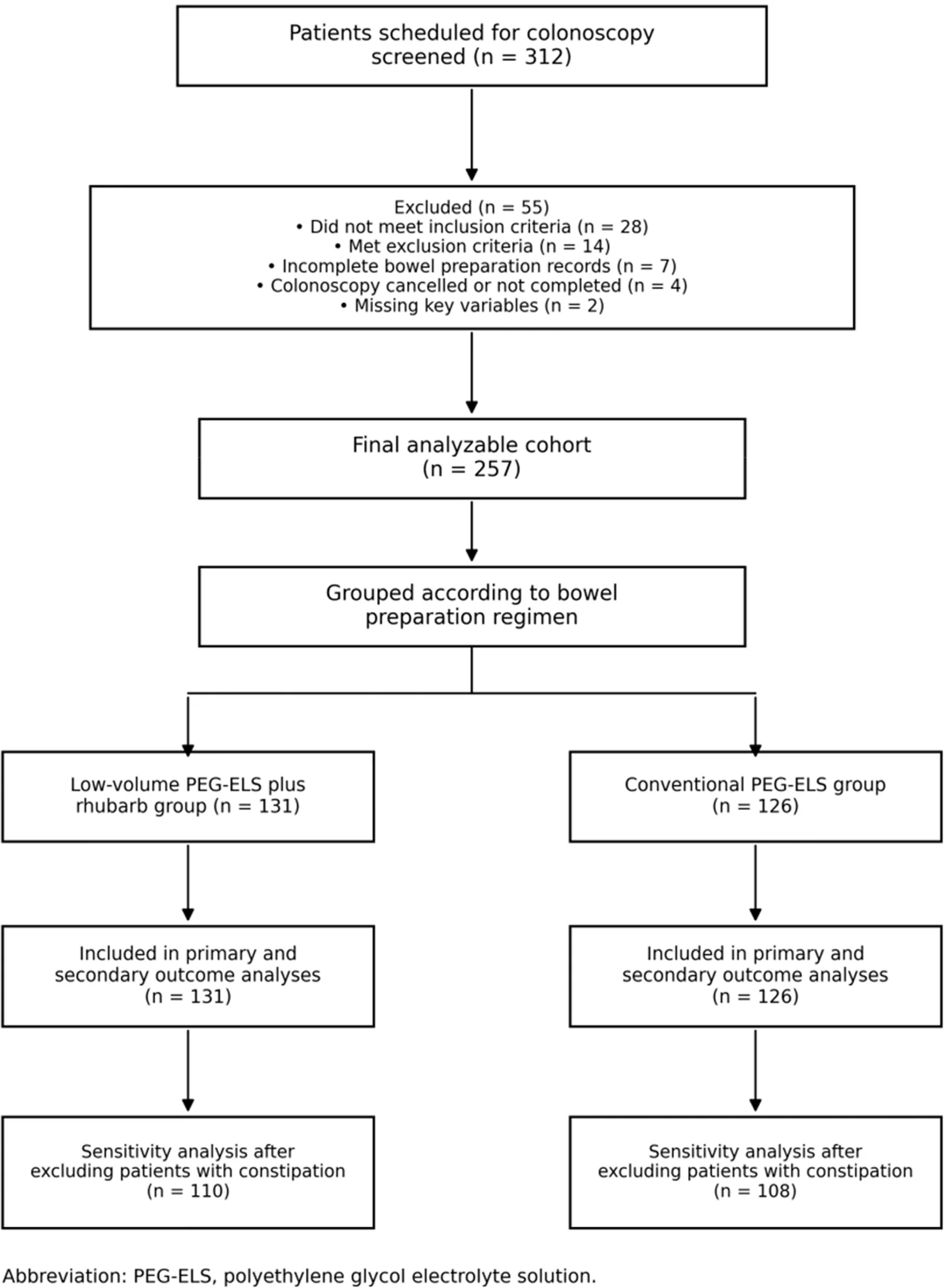
STROBE flowchart of patient screening and analysis. PEG-ELS, polyethylene glycol electrolyte solution.

Polyethylene glycol electrolyte solution (PEG-ELS), characterized by stable cleansing efficacy and minimal impact on water-electrolyte balance, ranks among the most widely used agents for pre-colonoscopy bowel preparation. Nevertheless, conventional PEG-ELS regimens require patients to ingest large volumes of liquid within a limited timeframe, frequently triggering nausea, vomiting, abdominal distension, abdominal pain, difficulty completing medication and sleep disturbance, thereby impairing patient compliance and satisfaction. In recent years, low-volume PEG regimens and strategies combining PEG with adjuvant laxatives have emerged as critical directions for bowel preparation optimization. Findings from multicenter randomized trials indicate that PEG-ELS combined with linaclotide constitutes an effective bowel preparation regimen for patients with chronic constipation prior to colonoscopy (2). Another study evaluating low-dose PEG combined with varying doses of linaclotide suggests that pairing laxatives with low-volume PEG may improve bowel cleansing efficacy and patient tolerance (3). Additional studies investigating PEG combined with lactulose or lubiprostone further validate the feasibility of low-volume PEG plus adjuvant laxatives as an optimized bowel preparation approach (4, 5). Collectively, these data demonstrate that reducing fluid intake burden while preserving cleansing quality has become a dominant trend in bowel preparation regimen refinement. Rhubarb is a commonly used purgative traditional Chinese medicinal herb, whose primary active ingredients include anthraquinone compounds such as rhein, emodin, aloe-emodin and sennosides. Modern pharmacological research posits that rhubarb and its anthraquinone constituents exert laxative effects by accelerating intestinal motility, regulating intestinal water and sodium absorption, modulating intestinal secretion and stimulating defecation reflexes (6, 7) Previous studies confirm that rhubarb or rhubarb-containing preparations exhibit certain efficacy in constipation management, providing pharmacological rationale and clinical translational potential for their application in bowel preparation (8). However, existing research on low-volume PEG bowel preparation predominantly focuses on Western medicine adjuvants, dietary modifications, carbohydrate clear liquid supplementation or optimized medication timing, with insufficient clinical evidence supporting rhubarb combined with low-volume PEG-ELS. Specifically, real-world data remain lacking regarding whether this regimen can maintain bowel cleansing quality despite reduced PEG-ELS dosage, mitigate gastrointestinal adverse reactions and enhance patient experience among general colonoscopy candidates. Most prior bowel preparation studies adopt BBPS scores, adequate bowel preparation rates or lesion detection rates as primary endpoints, while paying relatively limited attention to foam removal efficacy, time to first defecation, total bowel evacuation time, gastrointestinal tolerance, satisfaction and willingness to repeat colonoscopy. For patients requiring screening, re-examination or long-term follow-up, the clinical value of a bowel preparation regimen hinges not only on cleansing quality but also on patients' ability to complete preparation smoothly, their willingness to reuse the regimen and overall procedural experience. Against this backdrop, this retrospective single-center cohort study compares low-volume PEG-ELS plus rhubarb vs. conventional PEG-ELS regimens with respect to bowel preparation quality, defoaming efficacy, defecation kinetics, gastrointestinal adverse reactions, polyp detection rates, satisfaction and willingness to repeat colonoscopy, aiming to provide real-world evidence for developing colonoscopy bowel preparation regimens that integrate cleansing efficacy, gastrointestinal tolerance and favorable patient experience.

## 2 Materials and methods

### 2.1 General information

This retrospective single-center cohort study consecutively reviewed medical records of patients scheduled for elective colonoscopy who completed bowel preparation at Chongqing Kaizhou District People's Hospital between June 2023 and June 2024. Data were extracted from the hospital's electronic medical record system, endoscopy center examination records, bowel preparation assessment forms and pre- and post-procedure nursing evaluation documents, with all data anonymized prior to statistical analysis. Patients were stratified into two groups based on their actual bowel preparation regimens: the low-volume PEG-ELS plus rhubarb group (combined group) and the conventional PEG-ELS group (control group). The combined group received rhubarb plus 2 L PEG-ELS for bowel preparation, while the control group received 3 L PEG-ELS. Ultimately, 257 eligible patients were enrolled, including 131 in the combined group and 126 in the control group. Inclusion Criteria: 1. Aged ≥18 years; 2. Underwent elective colonoscopy at our hospital; 3. Completed the assigned bowel preparation regimen as instructed prior to examination; 4. Complete colonoscopy records available to retrieve total and segmental BBPS scores; 5. Key research variables including gastrointestinal adverse reactions, polyp detection, satisfaction and willingness to repeat colonoscopy fully documented. Exclusion Criteria: 1. Patients undergoing emergency colonoscopy; 2. Individuals with intestinal obstruction, gastrointestinal perforation, acute severe enteritis, active massive gastrointestinal hemorrhage or other conditions precluding routine bowel preparation or colonoscopy; 3. Prior colorectal resection or surgeries substantially altering colorectal anatomical structure; 4. Severe cardiac, hepatic or renal insufficiency or substantial risk of water-electrolyte disturbance; 5. Pregnant or lactating women; 6. Confirmed allergy to PEG-ELS or rhubarb preparations; 7. Failure to complete the prescribed bowel preparation, premature termination of colonoscopy, missing key outcome data or unverifiable medical records. Patients with a history of constipation were not excluded from the primary cohort. Constipation was retained because it is an important patient-related factor affecting bowel preparation quality and may modify the response to adjunctive laxatives. Therefore, constipation history was collected as a baseline variable and incorporated into the adjusted analyses. Baseline data collected included sex, age, body mass index (BMI), constipation history, diabetes history, prior abdominal surgery history, prior colonoscopy history, outpatient colonoscopy status and interval from bowel preparation to colonoscopy. These variables were used to describe baseline participant characteristics, evaluate intergroup comparability and serve as covariates adjusted for in multivariate regression analyses to control potential confounding bias. Cases with missing key outcome variables were excluded from final analyses; non-key variable discrepancies were cross-verified against original medical, endoscopy and nursing records to reduce information bias. This study complied with the principles outlined in the Declaration of Helsinki and received ethical approval from the Ethics Committee of Chongqing Kaizhou District People's Hospital. As a retrospective observational study without additional patient interventions and fully anonymized data, written informed consent was waived upon application. The study was designed, analyzed and reported in accordance with the STROBE (Strengthening the Reporting of Observational Studies in Epidemiology) statement for observational research. To ensure transparency, primary endpoints, critical secondary endpoints, covariates and sensitivity analysis strategies were pre-specified prior to data extraction. All outcomes were extracted per predefined definitions to avoid selective reporting based on statistical significance.

### 2.2 Methods

#### 2.2.1 Bowel preparation regimens

Control group (conventional PEG-ELS regimen): Compound polyethylene glycol electrolyte powder (Heshuang, 137.15 g per sachet, manufactured by Shenzhen Wanhe Pharmaceutical Co., Ltd.) was dissolved in warm boiled water to prepare 1 L solution per sachet per product instructions and hospital standard protocols for split oral administration. Patients ingested 1 L PEG-ELS between 20:00 and 22:00 on the evening preceding colonoscopy, followed by an additional 2 L between 08:00 and 10:00 on the morning of the examination.

Combined group (low-volume PEG-ELS plus rhubarb regimen): Patients ingested 1 L PEG-ELS between 20:00 and 22:00 the night before colonoscopy. At 08:00 on the morning of the procedure, patients first consumed decocted rhubarb solution followed by 1 L PEG-ELS administered in split doses per hospital protocols. Each patient received 9 g processed rhubarb brewed in approximately 100 mL warm boiled water for oral intake. Processed rhubarb decoction pieces were uniformly dispensed by the hospital pharmacy, with complete documentation of source, processing specifications, batch numbers and quality acceptance records per hospital Chinese medicinal herb quality control protocols. Medication orders, nursing records and bowel preparation logs were cross-checked during data extraction to verify dosage, administration timing and regimen completion.

All patients were encouraged to ambulate moderately during medication intake to facilitate intestinal evacuation. Standardized bowel preparation education was delivered to both groups per endoscopy center routine protocols. One day prior to colonoscopy, patients were instructed to consume a low-residue, low-fiber diet, avoiding seeded fruits, high-fiber vegetables, seaweed and dark-colored foods that interfere with intestinal visualization. Fasting was implemented per clinical instructions before the procedure, with fluid restriction enforced according to anesthesia modality and examination scheduling. Nursing staff instructed patients on PEG-ELS preparation, administration timing, ingestion rate, defecation monitoring and adverse reaction management protocols. Individualized medication guidance was provided by attending physicians for patients with hypertension, diabetes or long-term anticoagulant/antiplatelet therapy, balancing underlying disease management, examination objectives and endoscopic procedural risks. Patients presenting with severe abdominal pain, recurrent vomiting, dizziness, palpitations or suspected water-electrolyte disturbances during bowel preparation were assessed and managed by medical staff per endoscopy center standard operating procedures.

#### 2.2.2 Colonoscopy procedures

All patients underwent colonoscopy upon completion of bowel preparation. Pre-procedure nursing verification included patient identity, bowel preparation completion status, fasting and fluid restriction adherence, medical history, medication history and contraindication screening. Colonoscopies were performed by qualified endoscopists following hospital standardized protocols. Intraprocedural documentation included cecal intubation success, insertion time, withdrawal time, intestinal cleansing grade, foam/bubble interference and detection of intestinal lesions such as polyps. BBPS scores were assigned during withdrawal, with separate scores recorded for the right colon, transverse colon and left colon. Endoscopists determined biopsy or endoscopic treatment based on lesion size, morphology, location, patient comorbidities and endoscopic treatment indications upon identification of polyps or suspicious lesions. Polyp detection was documented per colonoscopy reports. Cases with premature examination termination due to severely inadequate bowel preparation, patient intolerance, technical limitations or other factors precluding evaluation of key outcomes were excluded from final analyses.

#### 2.2.3 Data collection

Uniformly trained researchers retrospectively extracted study data from electronic medical records, endoscopy center examination logs, bowel preparation assessment forms and nursing evaluation documents. Extracted data encompassed four categories: 1. Baseline data: sex, age, BMI, constipation history, diabetes history, prior abdominal surgery history, prior colonoscopy history, outpatient colonoscopy status, interval from bowel preparation to colonoscopy; 2. Bowel preparation and colonoscopy procedural data: preparation regimen, total PEG-ELS volume administered, rhubarb usage, total and segmental BBPS scores, defoaming score, bubble-clearance time, insertion time, withdrawal time, time to first defecation, total bowel evacuation time, polyp detection outcomes; 3. Gastrointestinal tolerance, compliance and patient experience data: nausea, vomiting, abdominal distension, abdominal pain and other gastrointestinal adverse reactions, completion of bowel preparation within the prescribed time window, self-reported difficulty completing the regimen, requirement for rescue preparation measures, patient satisfaction and willingness to reuse the identical bowel preparation regimen for future colonoscopy; 4. Safety data: documented severe bowel preparation-related adverse events, clinically apparent dehydration, severe abdominal pain requiring pharmacotherapy, aspiration events, emergency management secondary to bowel preparation, electrolyte disturbances requiring intervention. Definitions of time endpoints: Time to first defecation: interval from initial ingestion of morning bowel preparation medication to first defecation; Total bowel evacuation time: interval from initial ingestion of morning bowel preparation medication to passage of clear or near-clear effluent; Insertion time: duration from anal intubation to cecal/ileocecal valve arrival; Bubble-clearance time: cumulative time spent on irrigation and/or suction specifically for removing intraluminal bubbles during colonoscope insertion, recorded in seconds; Withdrawal time: duration from cecal/ileocecal valve withdrawal to anal exit, excluding therapeutic intervention time. Gastrointestinal adverse reactions were defined as documented episodes in nursing assessments, pre-procedure evaluation forms or bowel preparation observation logs. Satisfaction and willingness to repeat the regimen were abstracted from post-procedure nursing evaluations or follow-up records and coded as binary variables. Endoscopic pathology reports were further reviewed to supplement adenoma detection, advanced adenoma detection and right-sided colon adenoma detection data. Definitions of lesion detection rates: Adenoma detection rate: proportion of patients with at least one pathologically confirmed adenoma; Advanced adenoma: lesion measuring ≥10 mm in maximum diameter, or pathology revealing villous/tubulovillous components, or high-grade intraepithelial neoplasia; Right-sided colon adenoma: pathologically confirmed adenoma located in the cecum, ascending colon, hepatic flexure or transverse colon. For patients with multiple lesions, detection endpoints were analyzed at the patient level (positive if at least one qualifying lesion was identified).

#### 2.2.4 Scoring criteria

1. BBPS for bowel cleansing quality: The colon is divided into three segments (right colon, transverse colon, left colon), each scored 0–3 points, with a total score range of 0–9 points.

0 points: Abundant solid/semi-solid stool residue irremovable, precluding visualization of the entire segmental mucosa; 1 point: Mild-to-moderate semi-solid stool/turbid fluid residue, partially obscuring segmental mucosal visualization; 2 points: Trace small amounts of pasty stool/turbid fluid residue, enabling satisfactory segmental mucosal visualization; 3 points: Virtually no residual stool or turbid fluid, enabling excellent segmental mucosal visualization. Adequate bowel preparation was defined as a total BBPS score ≥6 with each segmental score ≥2 (9).

2. Defoaming score: Graded based on foam/bubble interference with endoscopic visualization documented during colonoscopy: 0 points: No bubbles, clear visualization; 1 point: Sparse bubbles covering ≤50% of intestinal lumen diameter, minimal visual interference; 2 points: Moderate bubbles covering >50% of intestinal lumen diameter, partial visual obstruction; 3 points: Dense bubbles filling the entire intestinal lumen, severe visual obstruction. Lower defoaming scores indicate milder foam/bubble interference and clearer endoscopic visualization (10).

#### 2.2.5 Quality control

To mitigate measurement bias, BBPS and defoaming scores were recorded exclusively during colonoscopic withdrawal, with researchers extracting data blind to original endoscopic scoring. Patient-reported outcomes were prioritized from standardized post-procedure nursing evaluations or follow-up logs; in cases of conflicting outcome documentation across multiple sources, the record closest to colonoscopy completion was adopted. A unified data extraction form was utilized to reduce information bias in retrospective data collection, with two independent researchers performing dual data entry and cross-verification. Discrepant records were re-reviewed against electronic medical, endoscopy and nursing logs; unresolved inconsistencies were adjudicated by a third researcher. Cases with missing key outcome variables were excluded from analyses; non-key variable gaps were supplemented via cross-referencing original clinical documentation. Age, sex, BMI and constipation history were incorporated as covariates in multivariate regression models to adjust primary and secondary outcomes for selection and confounding bias.

### 2.3 Statistical methods

Statistical analyses were performed using SPSS 26.0 software. The Shapiro–Wilk test was first used to assess the normality of continuous variables. Normally distributed data were expressed as mean ± standard deviation, and intergroup comparisons were conducted via independent-samples *t*-test with Welch's correction applied in cases of unequal variances. Non-normally distributed data were presented as median (interquartile range), and the Mann–Whitney *U*-test was adopted for between-group comparisons. Categorical variables were reported as case numbers (percentages) and compared using the chi-square test or Fisher's exact test as appropriate. Multivariate linear regression was used to analyze primary continuous outcomes, while multivariate logistic regression was performed for categorical outcomes. The models were primarily adjusted for age, sex, BMI and constipation history, with adjusted *β* coefficients or odds ratios (OR) alongside their 95% confidence intervals (CI) reported. Given the limited overall sample size and low event counts for several endpoints, covariates including diabetes history, prior abdominal surgery history, prior colonoscopy history, outpatient colonoscopy status, and the interval from bowel preparation to colonoscopy were only incorporated into propensity score models and sensitivity analyses to verify result robustness. To address the core research question of whether the low-volume combined regimen could preserve bowel cleansing efficacy while reducing the total PEG-ELS dosage, exploratory non-inferiority analyses were conducted for the adequate bowel preparation rate and total BBPS score. The pre-specified non-inferiority margin was −10% for the adequate bowel preparation rate and −1 point for the total BBPS score. The −10% margin for adequate bowel preparation rate was selected with reference to widely accepted clinical thresholds adopted in previous non-inferiority trials of low-volume PEG bowel preparation, tailored to the exploratory real-world design of this study. The −1-point cutoff for total BBPS score was determined based on the clinical interpretability of the BBPS scale and the acceptable range of changes in individual segment scores. The defoaming score was treated as an ordinal variable. The primary analyses comprised rank-sum tests and regression after rank transformation, with ordered logistic regression implemented for sensitivity validation. Variance inflation factors (VIF < 5) were calculated to evaluate multicollinearity in regression models, and the Hosmer-Lemeshow test was used to assess model goodness-of-fit. To evaluate the robustness of the findings, sensitivity analyses were carried out using 1:1 propensity score matching (PSM) and inverse probability of treatment weighting (IPTW). The propensity score model included the following covariates: age, sex, BMI, constipation history, diabetes history, prior abdominal surgery history, prior colonoscopy history, outpatient colonoscopy status, and the interval from bowel preparation to colonoscopy. For PSM, nearest-neighbor matching without replacement was performed with a caliper width set to 0.2 times the standard deviation of the logit propensity score; standardized mean difference (SMD) < 0.10 was defined as the criterion for adequate covariate balance. Stabilized weights with robust standard error estimation were utilized for IPTW. Propensity score analyses reported post-matching sample sizes as well as SMD balance before matching, after matching, and following IPTW weighting. Separate effect estimates (*β* for continuous outcomes, OR for categorical outcomes) with corresponding 95% CIs were presented under both the PSM and IPTW frameworks. All statistical tests were two-tailed, and a *P* value less than 0.05 was defined as the threshold for statistical significance. In addition, considering that patients with constipation might derive greater benefit from the stimulant laxative effect of rhubarb, a *post hoc* exploratory subgroup analysis was performed among patients with a documented history of constipation. Within this subgroup, total BBPS score, adequate bowel preparation rate, defoaming score, insertion time, time to first defecation, and total bowel evacuation time were compared between the two preparation regimens. BBPS and defoaming scores were compared using the Mann–Whitney *U*-test. Continuous time variables were compared using the independent-samples *t*-test after assessment of normality and homogeneity of variance, and categorical variables were compared using Fisher's exact test when appropriate. Because constipation subtype was not routinely documented in the retrospective records, this analysis was based on a history of constipation and could not specifically identify patients with slow-transit constipation. This subgroup analysis was considered exploratory.

## 3 Results

### 3.1 Patient screening flow and study population

A total of 312 medical records of patients scheduled for colonoscopy were screened, with 55 excluded: 28 failing to meet inclusion criteria, 14 satisfying exclusion criteria, 7 with incomplete bowel preparation documentation, 4 with canceled/unfinished colonoscopy, and 2 with missing key variables (Figure 1). The final analyzable cohort included 257 patients, with 131 receiving low-volume PEG-ELS plus rhubarb and 126 receiving conventional PEG-ELS. All enrolled participants possessed complete primary outcome data and were included in primary and secondary endpoint analyses. For sensitivity analysis excluding patients with constipation history, 39 individuals were removed, yielding a subcohort of 218 patients (110 combined group, 108 control group) for repeated analyses.

### 3.2 Baseline characteristic comparison between groups

No statistically significant intergroup differences were observed in sex distribution, age, BMI, constipation history, diabetes history, prior abdominal surgery history, prior colonoscopy history, proportion of outpatient colonoscopy or interval from bowel preparation to colonoscopy (all *P* > 0.05), indicating overall balanced baseline characteristics between the two groups (Table 1).

**Table 1:** Comparison of baseline characteristics between the two groups.

Variable	Combined group (*n* = 131)	Control group (*n* = 126)	Statistic	*P* value
Male, *n* (%)	68 (51.91)	62 (49.21)	*χ*^2^ = 0.188	0.665
Age (years, mean ± SD)	54.37 ± 11.30	55.82 ± 10.45	*t* = −1.062	0.289
BMI (kg/m^2^, mean ± SD)	23.86 ± 3.17	24.11 ± 2.94	*t* = −0.654	0.514
Constipation history, *n* (%)	21 (16.03)	18 (14.29)	χ^2^ = 0.152	0.697
Diabetes history, *n* (%)	15 (11.45)	17 (13.49)	χ^2^ = 0.246	0.620
Previous abdominal surgery, *n* (%)	22 (16.79)	24 (19.05)	χ^2^ = 0.221	0.638
Previous colonoscopy history, *n* (%)	38 (29.01)	34 (26.98)	χ^2^ = 0.133	0.715
Outpatient colonoscopy, *n* (%)	111 (84.73)	103 (81.75)	χ^2^ = 0.406	0.524
Interval from bowel preparation to colonoscopy (h, mean ± SD)	4.81 ± 1.08	4.96 ± 1.16	*t* = −1.071	0.285

BMI, body mass index. Continuous variables were compared using the independent-samples *t*-test, and categorical variables were compared using the chi-square test.

### 3.3 Bowel cleansing quality comparison

No statistically significant intergroup difference was detected in total BBPS scores (*P* = 0.145), nor in segmental BBPS scores for the right, transverse and left colon (all *P* > 0.05). Defining adequate bowel preparation as total BBPS ≥ 6 with all segmental scores ≥2, the adequate preparation rate reached 94.66% (124/131) in the combined group vs. 89.68% (113/126) in the control group, with no statistically significant difference (*P* = 0.137) (Table 2, Figure 2). Exploratory non-inferiority analysis demonstrated that the adequate preparation rate was 4.98% higher in the combined group (95%CI: −1.54% to 11.50%), with the lower CI bound exceeding the pre-specified non-inferiority margin of −10%. The adjusted *β* for total BBPS score was 0.17 (95%CI: −0.05 to 0.40), whose lower bound surpassed the −1-point non-inferiority margin (Table 3, Figure 3).

**Table 2:** Comparison of bowel cleansing quality.

Outcome	Combined group (*n* = 131)	Control group (*n* = 126)	Statistic	*P* value
BBPS total score, M (IQR)	8 (7, 8)	8 (7, 8)	*U* = 9,074	0.145
Right-colon score, M (IQR)	2 (2, 3)	2 (2, 3)	*U* = 8,385	0.804
Transverse-colon score, M (IQR)	3 (2, 3)	3 (2, 3)	*U* = 8,554	0.552
Left-colon score, M (IQR)	3 (2, 3)	3 (2, 3)	*U* = 8,958	0.162
Adequate bowel preparation, *n* (%)	124 (94.66)	113 (89.68)	χ^2^ = 2.214	0.137

BBPS, Boston Bowel Preparation Scale; IQR, interquartile range. BBPS segment scores were compared using the Mann–Whitney *U*-test. Adequate bowel preparation was defined as a BBPS total score ≥6 with each segment score ≥2.

**Figure 2:**
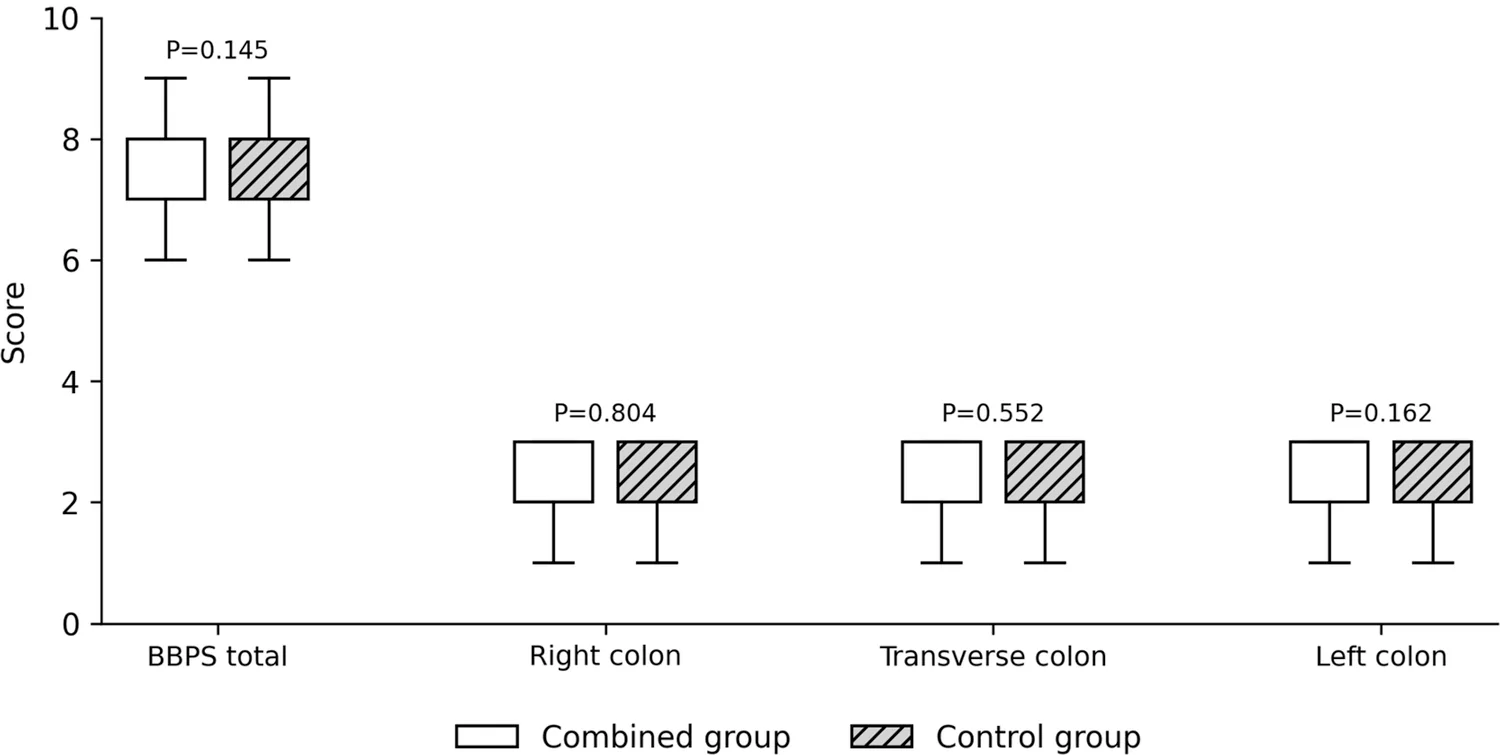
Comparison of BBPS scores between the two groups. Boxplots show medians, interquartile ranges, whiskers, and outliers. *P* values were calculated using the Mann–Whitney *U*-test.

**Table 3:** Exploratory non-inferiority analysis of primary cleansing-quality outcomes.

Outcome	Effect estimate	95%CI	Prespecified non-inferiority margin
Difference in adequate bowel preparation rate (combined group—control group)	4.98%	−1.54%–11.50%	−10%
Adjusted *β* for BBPS total score	0.17 points	−0.05–0.40	−1 points

**Figure 3:**
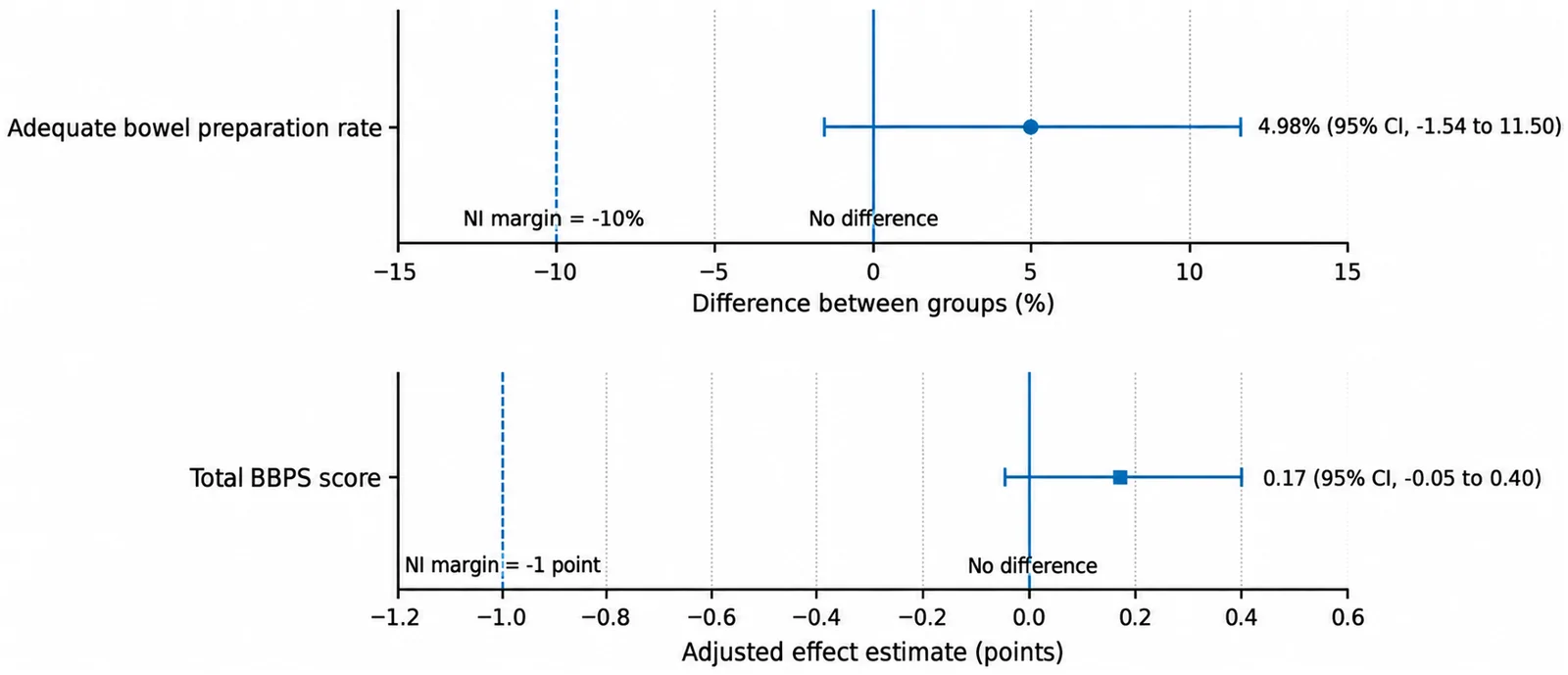
Exploratory non-inferiority analysis of primary bowel-cleansing outcomes. The between-group difference in adequate bowel preparation rate and the adjusted effect estimate for total BBPS score are shown with 95% confidence intervals. The dashed vertical lines indicate the prespecified non-inferiority margins of −10% for adequate bowel preparation rate and −1 point for total BBPS score. BBPS, Boston Bowel Preparation Scale; CI, confidence interval; NI, non-inferiority.

### 3.4 Defoaming score, colonoscopy procedural indicators and lesion detection rate comparison

The combined group had a significantly lower defoaming score than the control group [1 (0, 1) vs. 1 (1, 1), *P* = 0.012] (Figure 4). Bubble-clearance time was also shorter in the combined group (18.72 ± 7.64 s vs. 27.35 ± 9.12 s, *P* < 0.001). However, no statistically significant differences were observed in insertion time or withdrawal time between the two groups (both *P* > 0.05). Similarly, polyp detection rate, adenoma detection rate, advanced adenoma detection rate, and right-sided adenoma detection rate did not differ significantly between groups (all *P* > 0.05) (Table 4).

**Figure 4:**
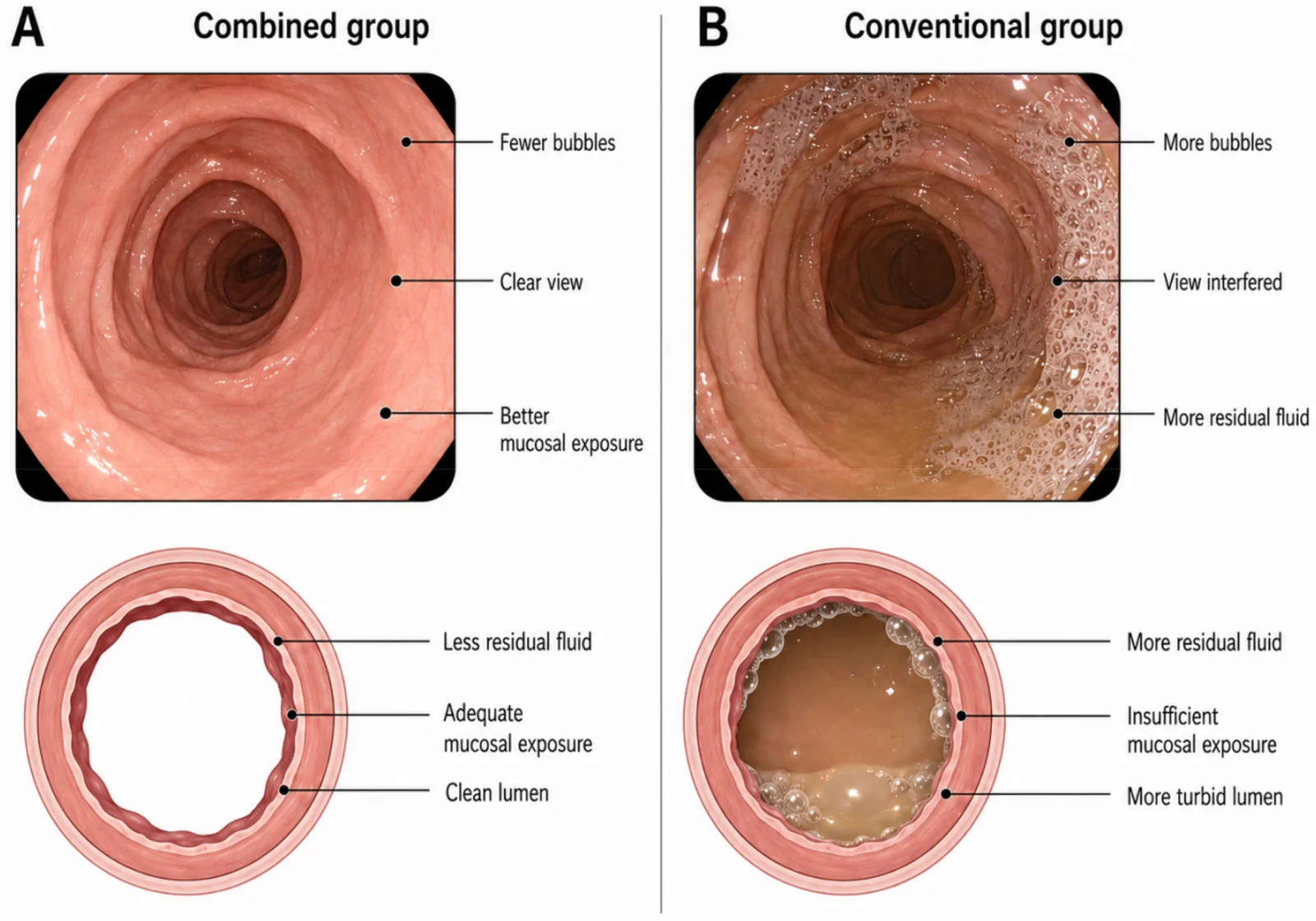
Schematic illustration of colonoscopic visual-field differences between low-volume PEG-ELS plus rhubarb and conventional PEG-ELS. **(A)** The combined group is schematically illustrated as having fewer bubbles, less residual fluid, a clearer endoscopic view, and more adequate mucosal exposure, the lower cross-sectional intestinal-lumen diagram indicates a cleaner lumen and better mucosal visualization. **(B)** The conventional group is schematically illustrated as having more bubbles and residual fluid, partial visual-field interference, and relatively insufficient mucosal exposure.

**Table 4:** Comparison of defoaming score, colonoscopy-related procedural indicators, and lesion detection rates.

Indicator	Combined group (*n* = 131)	Control group (*n* = 126)	Effect estimate	Statistic	*P* value
Defoaming score, M (IQR)	1 (0, 1)	1 (1, 1)	–	*U* = 6,896	0.012
Insertion time (min, mean ± SD)	6.84 ± 2.16	7.13 ± 2.38	−0.29(−0.85–0.27)	*t* = −1.029	0.304
Bubble-clearance time, s, mean ± SD	18.72 ± 7.64	27.35 ± 9.12	−8.63(−10.70–6.56)	*t* = −8.207	<0.001
Withdrawal time (min, mean ± SD)	8.27 ± 2.41	8.59 ± 2.73	−0.32(−0.95–0.31)	*t* = −0.996	0.320
Polyp detection, *n* (%)	52 (39.69)	47 (37.30)	–	χ^2^ = 0.155	0.694
Adenoma detection, *n* (%)	39 (29.77)	36 (28.57)	1.06 (0.62–1.82)	χ^2^ = 0.045	0.833
Advanced adenoma detection, *n* (%)	8 (6.11)	7 (5.56)	1.11 (0.39–3.14)	Fisher exact	1.000
Right-sided adenoma detection, *n* (%)	18 (13.74)	16 (12.70)	1.10 (0.53–2.26)	χ^2^ = 0.061	0.805

IQR, interquartile range; OR, odds ratio; CI, confidence interval. Defoaming score was an ordinal variable and was compared using the Mann–Whitney *U*-test. Insertion time and withdrawal time were compared using the independent-samples *t*-test. Categorical variables were compared using the chi-square test or Fisher exact test. Adenoma detection, advanced adenoma detection, and right-sided adenoma detection were analyzed at the patient level, with positivity defined as detection of at least one corresponding lesion in the same patient.

### 3.5 Bowel preparation response time comparison

Time to first defecation was significantly shorter in the combined group (38.42 ± 12.71 min vs. 51.86 ± 15.97 min; mean difference = −13.44 min, 95%CI: −16.99 to −9.88; *P* < 0.001). Total bowel evacuation time was also markedly reduced in the combined group (182.54 ± 41.30 min vs. 218.79 ± 49.64 min; mean difference = −36.25 min, 95%CI: −47.45 to −25.05; *P* < 0.001) (Table 5).

**Table 5:** Comparison of bowel preparation response times.

Indicator	Combined group (*n* = 131)	Control group (*n* = 126)	Mean difference (95% CI)	Statistic	*P* value
Time to first defecation (min, mean ± SD)	38.42 ± 12.71	51.86 ± 15.97	−13.44(−16.99 to −9.88)	Welch *t* = −7.447	<0.001
Time to bowel evacuation (min, mean ± SD)	182.54 ± 41.30	218.79 ± 49.64	−36.25(−47.45 to −25.05)	*t* = −6.374	<0.001

CI, confidence interval. Time variables were compared using the independent-samples *t*-test; Welch-corrected *t*-test was used when variances were unequal for time to first defecation.

### 3.6 Gastrointestinal adverse reaction comparison

The combined group demonstrated superior gastrointestinal tolerance. Nausea incidence was lower in the combined group (23.66% vs. 37.30%, *P* = 0.017), vomiting incidence reduced (9.16% vs. 19.05%, *P* = 0.022), and abdominal distension incidence decreased (32.82% vs. 46.03%, *P* = 0.030). No statistically significant intergroup difference was observed in abdominal pain incidence (21.37% vs. 25.40%, *P* = 0.446). The overall incidence of any gastrointestinal adverse reaction was substantially lower in the combined group (62.60% vs. 84.13%, *P* < 0.001) (Table 6).

**Table 6:** Comparison of gastrointestinal adverse reactions.

Outcome	Combined group (*n* = 131)	Control group (*n* = 126)	Statistic	*P* value
Any gastrointestinal adverse reaction, *n* (%)	82 (62.60)	106 (84.13)	χ^2^ = 15.161	<0.001
Nausea, *n* (%)	31 (23.66)	47 (37.30)	χ^2^ = 5.651	0.017
Vomiting, *n* (%)	12 (9.16)	24 (19.05)	χ^2^ = 5.212	0.022
Abdominal distension, *n* (%)	43 (32.82)	58 (46.03)	χ^2^ = 4.696	0.030
Abdominal pain, *n* (%)	28 (21.37)	32 (25.40)	χ^2^ = 0.581	0.446

Categorical variables were compared using the chi-square test or Fisher exact test.

### 3.7 Patient-reported experience comparison

Patient satisfaction was significantly higher in the combined group (86.26% vs. 73.02%, *P* = 0.008). Willingness to reuse the identical bowel preparation regimen for future colonoscopy was also greater in the combined group (82.44% vs. 69.05%, *P* = 0.012) (Table 7).

**Table 7:** Comparison of patient-reported experience.

Outcome	Combined group (*n* = 131)	Control group (*n* = 126)	Statistic	*P* value
Satisfaction, *n* (%)	113 (86.26)	92 (73.02)	χ^2^ = 6.980	0.008
Willingness to reuse the same regimen, *n* (%)	108 (82.44)	87 (69.05)	χ^2^ = 6.296	0.012

Data are presented as *n* (%), and between-group comparisons were performed using the chi-square test.

### 3.8 Multivariate linear regression analysis of continuous outcomes

Multivariate linear regression adjusting for age, sex, BMI and constipation history was performed to control potential confounding factors, with bowel preparation regimen designated as the primary independent variable. No significant association was identified between combined regimen administration and total BBPS score (adjusted *β* = 0.17, 95%CI: −0.05∼0.40, *P* = 0.136). The combined regimen independently correlated with lower defoaming scores, shortened time to first defecation and shortened total bowel evacuation time (all *P* < 0.05) (Table 8).

**Table 8:** Multivariable linear regression analysis of continuous outcomes.

Outcome	adjusted β	95%CI	*P* value
BBPS total score	0.17	−0.05–0.40	0.136
Defoaming score	−0.20	−0.37 to −0.03	0.020
Time to first defecation, min	−13.20	−16.72 to −9.68	<0.001
Time to bowel evacuation, min	−36.66	−47.91 to −25.42	<0.001

The control group was used as the reference. Adjusted β represents the adjusted mean difference or rank-transformed effect estimate for the combined group relative to the control group. Models were adjusted for age, sex, BMI, and constipation history.

### 3.9 Multivariate logistic regression analysis of categorical outcomes

Multivariate logistic regression analysis was further performed to adjust for potential confounding factors, with adjustments made for age, sex, BMI and constipation history. After adjustment, no significant association was detected between bowel preparation regimen and the adequate bowel preparation rate (adjusted OR = 2.00, 95%CI: 0.76–5.23, *P* = 0.160). The combined regimen was correlated with a lower risk of any gastrointestinal adverse reaction (adjusted OR = 0.30, 95%CI: 0.17–0.56, *P* < 0.001), as well as reduced risks of nausea, vomiting and abdominal distension (all *P* < 0.05). No significant correlation was found between bowel preparation regimen and polyp detection rate (adjusted OR = 1.11, 95%CI: 0.66–1.84, *P* = 0.696). In addition, the combined regimen was associated with higher patient satisfaction (adjusted OR = 2.33, 95%CI: 1.23–4.43, *P* = 0.010) and greater willingness to reuse the identical preparation regimen (adjusted OR = 2.19, 95%CI: 1.21–3.97, *P* = 0.010) (Table 9, Figure 5).

**Table 9:** Multivariable logistic regression analysis of categorical outcomes.

Outcome	Adjusted OR	95%CI	*P* value
Adequate bowel preparation	2.00	0.76–5.23	0.160
Any gastrointestinal adverse reaction	0.30	0.17–0.56	<0.001
Nausea	0.50	0.29–0.87	0.014
Vomiting	0.42	0.20–0.88	0.023
Abdominal distension	0.58	0.35–0.97	0.038
Abdominal pain	0.80	0.45–1.45	0.467
Polyp detection	1.11	0.66–1.84	0.696
Satisfaction	2.33	1.23–4.43	0.010
Willingness to reuse the same regimen	2.19	1.21–3.97	0.010
Adenoma detection	1.08	0.62–1.88	0.785
Advanced adenoma detection	1.13	0.39–3.29	0.823
Right-sided adenoma detection	1.11	0.53–2.33	0.782

OR, odds ratio; CI, confidence interval. Adjusted ORs indicate the odds of each outcome in the combined group relative to the control group. Models were adjusted for age, sex, BMI, and constipation history. For adverse reactions, OR < 1 indicates a lower risk in the combined group; for satisfaction and willingness to undergo the same regimen again, OR > 1 indicates a higher probability in the combined group.

**Figure 5:**
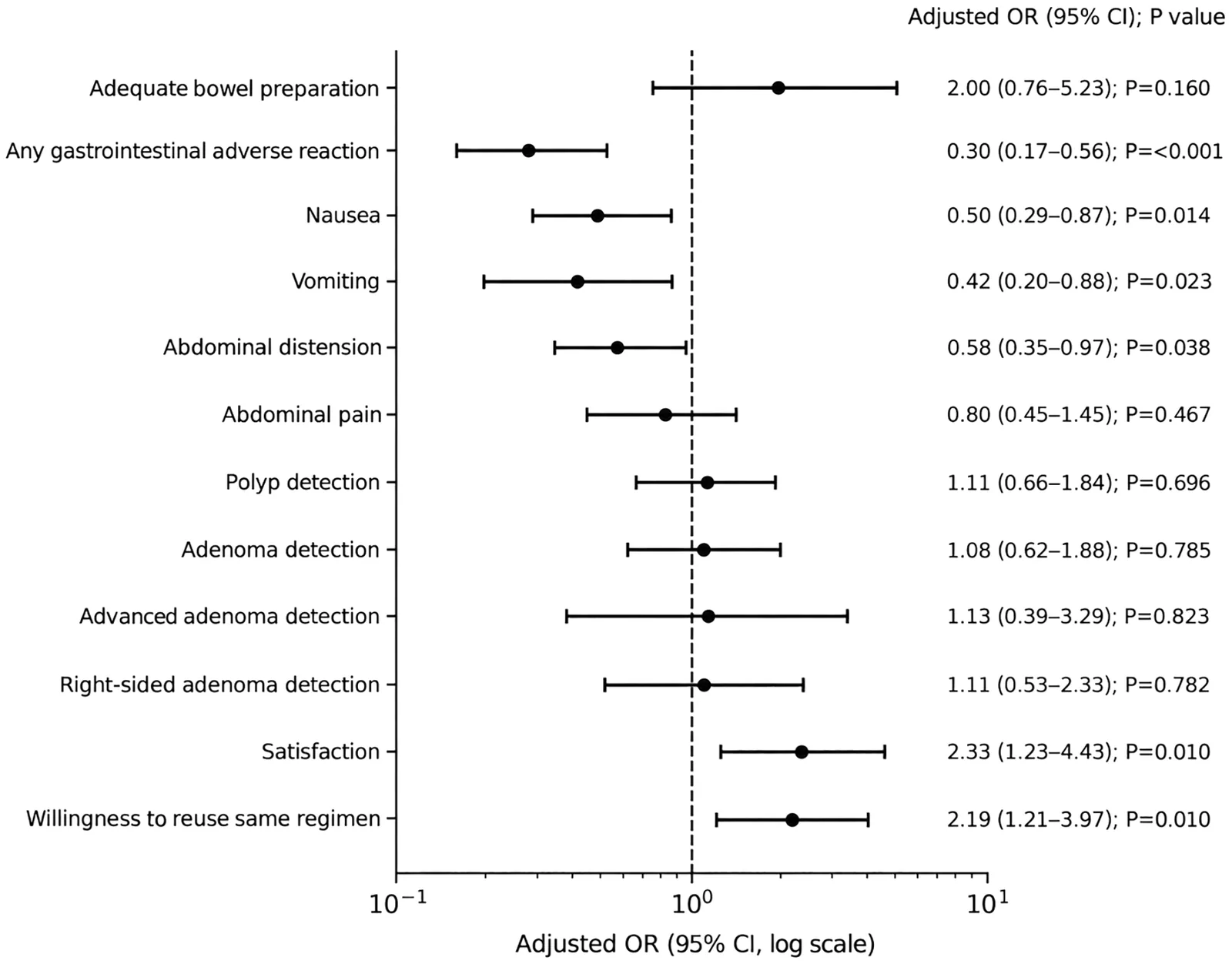
Adjusted OR forest plot. OR, odds ratio; CI, confidence interval. Adjusted ORs indicate the odds of each outcome in the combined group relative to the control group.

### 3.10 Propensity score and sensitivity analyses

A propensity score model was established incorporating age, sex, BMI, constipation history, diabetes history, prior abdominal surgery history, prior colonoscopy history, outpatient colonoscopy status, and the interval from bowel preparation to colonoscopy. After 1:1 nearest-neighbor matching without replacement, 120 matched patient pairs were generated (120 patients in the combined group and 120 patients in the control group). The standardized mean differences (SMDs) of all included covariates were less than 0.10 post-matching, indicating well-balanced baseline characteristics between the two groups; all covariates also yielded SMD values below 0.10 following inverse probability of treatment weighting (IPTW) (Table 10, Figure 6). Within both the propensity score matching (PSM) sample and IPTW-weighted sample, no significant intergroup differences were identified in total Boston Bowel Preparation Scale (BBPS) scores or adequate bowel preparation rates. The combined regimen was associated with lower defoaming scores, shorter time to first defecation and shorter total bowel evacuation time. The effect directions for any gastrointestinal adverse reaction, patient satisfaction and willingness to reuse the identical regimen remained consistent with primary analyses. No statistically significant intergroup differences were observed in polyp detection rate, adenoma detection rate, advanced adenoma detection rate or right-sided colon adenoma detection rate across PSM and IPTW analyses (Tables 11, 12). Sensitivity analysis was conducted after excluding patients with a history of constipation, enrolling a total of 218 subjects (110 patients in the combined group and 108 patients in the control group). The results of the sensitivity analysis were consistent with those of the primary analysis: there were no statistically significant differences in total BBPS scores or adequate bowel preparation rates between the two groups (all *P* > 0.05). The combined group had lower defoaming scores (*P* = 0.006), shorter time to first defecation and shorter total bowel evacuation time (all *P* < 0.001), as well as a lower overall incidence of gastrointestinal adverse reactions (*P* = 0.003). Higher levels of patient satisfaction and greater willingness to reuse the preparation regimen were also observed in the combined group (*P* = 0.023 and *P* = 0.018, respectively) (Table 13).

**Table 10:** Covariate balance before and after propensity score matching and after IPTW weighting.

Variable	SMD before matching	SMD after 1:1 PSM	SMD after IPTW
Age	0.133	0.028	0.021
Male	0.054	0.017	0.012
BMI	0.082	0.031	0.026
Constipation history	0.049	0.019	0.015
Diabetes history	0.062	0.024	0.018
Previous abdominal surgery	0.059	0.020	0.017
Previous colonoscopy history	0.045	0.018	0.014
Outpatient colonoscopy	0.079	0.026	0.020
Interval from bowel preparation to colonoscopy	0.134	0.036	0.029

SMD, standardized mean difference; PSM, propensity score matching; IPTW, inverse probability of treatment weighting. PSM was performed using 1:1 nearest-neighbor matching without replacement with a caliper of 0.2 times the standard deviation of the logit propensity score. IPTW used stabilized weights. SMD < 0.10 indicates good covariate balance.

**Figure 6:**
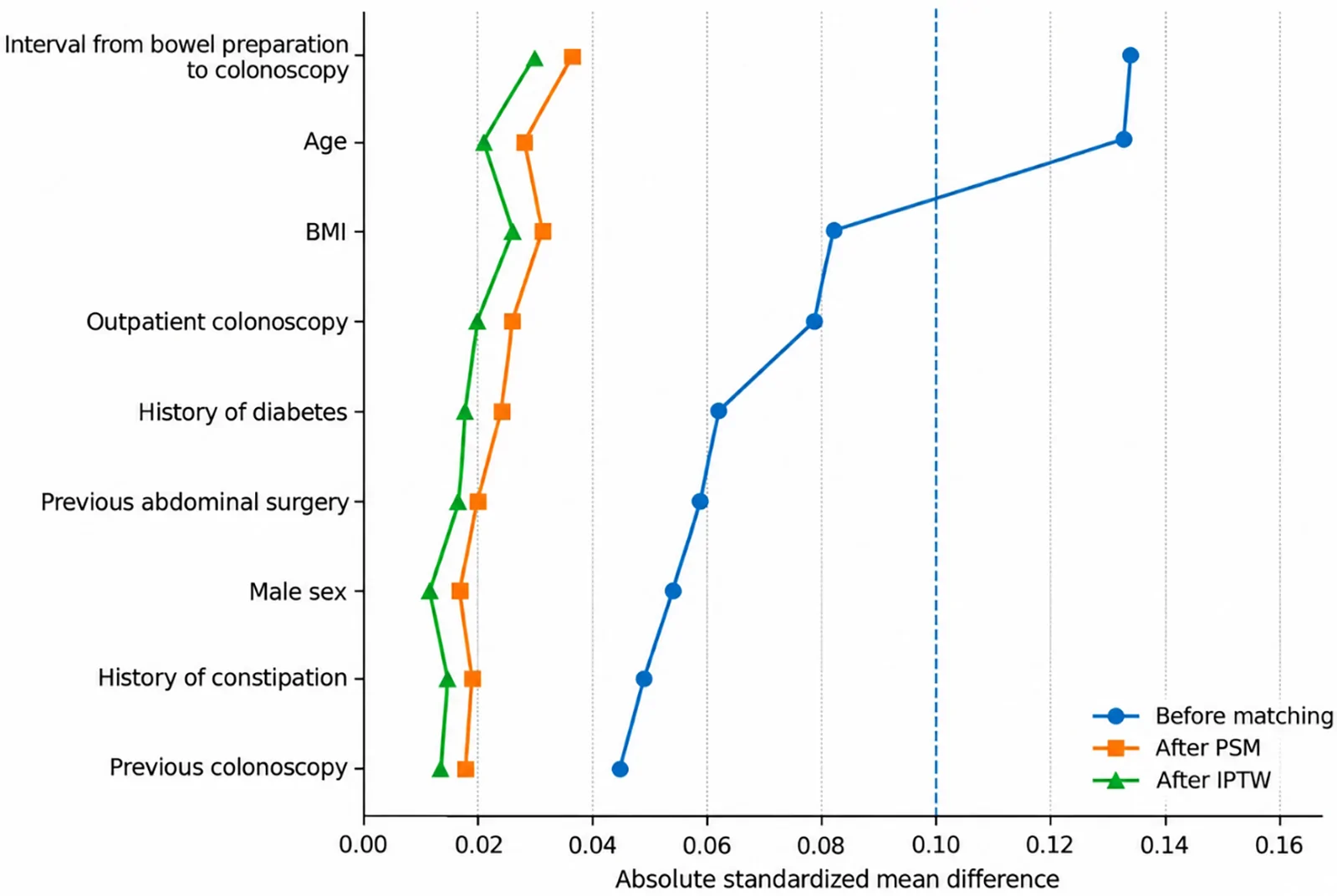
Covariate balance before matching, after propensity score matching, and after inverse probability of treatment weighting. Absolute standardized mean differences were used to assess covariate balance between the combined group and the control group. The dashed vertical line indicates the prespecified balance threshold of 0.10. PSM, propensity score matching; IPTW, inverse probability of treatment weighting; SMD, standardized mean difference; BMI, body mass index.

**Table 11:** Effect estimates for major continuous outcomes after PSM and IPTW analyses.

Outcome	β after PSM (95% CI)	*P* value	β after IPTW (95% CI)	*P* value
BBPS total score	0.15(−0.09–0.39)	0.219	0.16(−0.06–0.38)	0.151
Defoaming score	−0.21(−0.37 to −0.05)	0.010	−0.20(−0.36 to −0.04)	0.015
Time to first defecation, min	−13.08(−16.73 to −9.44)	<0.001	−13.32(−16.91 to −9.73)	<0.001
Time to bowel evacuation, min	−35.21(−47.04 to −23.38)	<0.001	−36.11(−47.44 to −24.78)	<0.001

β values represent effect estimates for the combined group relative to the control group. The PSM model was based on the 1:1 nearest-neighbor matched sample, and the IPTW model used stabilized weights with robust standard errors.

**Table 12:** Effect estimates for major categorical outcomes after PSM and IPTW analyses.

Outcome	OR after PSM (95% CI)	*P* value	OR after IPTW (95% CI)	*P* value
Adequate bowel preparation	1.78 (0.65–4.86)	0.258	1.86 (0.71–4.89)	0.207
Any gastrointestinal adverse reaction	0.31 (0.16–0.60)	<0.001	0.32 (0.18–0.57)	<0.001
Nausea	0.52 (0.29–0.95)	0.033	0.51 (0.29–0.89)	0.018
Vomiting	0.44 (0.19–0.99)	0.047	0.43 (0.20–0.91)	0.028
Abdominal distension	0.59 (0.34–1.02)	0.060	0.57 (0.34–0.96)	0.035
Polyp detection	1.08 (0.63–1.86)	0.771	1.10 (0.67–1.81)	0.704
Adenoma detection	1.05 (0.59–1.88)	0.864	1.07 (0.62–1.85)	0.811
Advanced adenoma detection	1.12 (0.37–3.40)	0.842	1.10 (0.38–3.22)	0.858
Right-sided adenoma detection	1.09 (0.51–2.35)	0.821	1.12 (0.54–2.34)	0.758
Satisfaction	2.21 (1.12–4.38)	0.023	2.27 (1.19–4.33)	0.013
Willingness to reuse the same regimen	2.09 (1.09–4.00)	0.026	2.13(1.14–3.98)	0.018

OR, odds ratio; CI, confidence interval. The PSM model was based on the 1:1 nearest-neighbor matched sample, and the IPTW model used stabilized weights. OR < 1 indicates a lower risk in the combined group, and OR > 1 indicates a higher probability in the combined group.

**Table 13:** Additional sensitivity analysis restricted to patients without a history of constipation.

Outcome	Combined group (*n* = 110)	Control group (*n* = 108)	Statistic	*P* value
BBPS total score, M (IQR); mean ± SD	8 (7, 8); 7.68 ± 0.88	8 (7, 8); 7.53 ± 0.98	*U* = 6,466	0.233
Adequate bowel preparation, *n* (%)	104 (94.55)	98 (90.74)	χ^2^ = 1.160	0.281
Defoaming score, M (IQR); mean ± SD	1 (0, 1); 0.73 ± 0.70	1 (1, 1); 0.96 ± 0.64	*U* = 4,778	0.006
Time to first defecation, min	38.27 ± 12.12	51.69 ± 16.28	Welch *t* = −6.895	<0.001
Time to bowel evacuation, min	183.00 ± 42.48	217.28 ± 48.79	t = −5.535	<0.001
Any gastrointestinal adverse reaction, *n* (%)	74 (67.27)	91 (84.26)	χ^2^ = 8.545	0.003
Satisfaction, *n* (%)	95 (86.36)	80 (74.07)	χ^2^ = 5.198	0.023
Willingness to reuse the same regimen, *n* (%)	93 (84.55)	77 (71.30)	χ^2^ = 5.571	0.018

After excluding patients with a history of constipation, BBPS scores and defoaming scores were compared using the Mann–Whitney *U*-test, time variables were compared using the independent-samples *t*-test, and categorical variables were compared using the chi-square test or Fisher exact test. This analysis was performed solely as a sensitivity analysis to assess the robustness of the primary findings and did not represent an exclusion criterion for the primary study cohort. Patients with constipation remained included in all primary analyses.

### 3.11 Exploratory subgroup analysis in patients With constipation

A total of 39 patients with a documented history of constipation were included in the exploratory subgroup analysis, including 21 patients in the combined group and 18 patients in the control group. The combined group showed significantly shorter time to first defecation and total bowel evacuation time than the control group (both *P* < 0.05). No statistically significant differences were observed in total BBPS score, adequate bowel preparation rate, defoaming score, or insertion time between the two groups (all *P* > 0.05) (Table 14).

**Table 14:** Exploratory analysis of bowel preparation outcomes in patients with a history of constipation.

Outcome	Combined group (*n* = 21)	Control group (*n* = 18)	Statistic	*P* value
BBPS total score, M (IQR)	8 (7, 8)	7 (7, 8)	*U* = 219.000	0.375
Adequate bowel preparation, *n* (%)	20 (95.24)	15 (83.33)	Fisher exact	0.318
Defoaming score, M (IQR)	1 (0, 1)	1 (0, 1)	*U* = 192.500	0.926
Insertion time, min, mean ± SD	6.18 ± 2.68	7.74 ± 2.64	*t* = −1.825	0.076
Time to first defecation, min, mean ± SD	39.19 ± 15.77	52.83 ± 14.30	*t* = −2.811	0.008
Total bowel evacuation time, min, mean ± SD	180.14 ± 35.27	227.89 ± 55.09	*t* = −3.270	0.002

BBPS, Boston Bowel Preparation Scale; IQR, interquartile range; SD, standard deviation. Adequate bowel preparation was defined as a total BBPS score ≥6 with each segmental score ≥2. BBPS and defoaming scores were compared using the Mann–Whitney *U*-test; continuous variables were compared using the independent-samples *t*-test; Fisher's exact test was used for adequate bowel preparation. This was a *post hoc* exploratory subgroup analysis based on documented constipation history. Constipation subtype, including slow-transit constipation, was not routinely recorded.

### 3.12 Supplementary analysis of compliance and severe safety events

A higher proportion of patients in the combined group completed bowel preparation within the prescribed time window (92.37% vs. 84.13%, *P* = 0.039), with fewer patients reporting difficulty completing the regimen (17.56% vs. 32.54%, *P* = 0.005) (Table 15). No severe bowel preparation-related adverse events were documented in either group, and no colonoscopies were prematurely terminated secondary to inadequate preparation. No intergroup statistically significant differences were observed in clinically apparent dehydration, severe abdominal pain requiring pharmacotherapy, aspiration events, bowel preparation-related emergency intervention or electrolyte disturbances requiring treatment (Table 16). Review of electronic medical records captured only short-term clinical safety events documented in medical and nursing logs. Pre- and post-procedure paired electrolyte and renal function testing (serum potassium, sodium, creatinine, blood urea nitrogen, estimated glomerular filtration rate) was not routine within the endoscopy center within 24–72 h post-colonoscopy, complete paired laboratory safety data were unavailable. Therefore, safety outcomes reflect only short-term clinical events identifiable in medical records and cannot substitute systematic laboratory safety evaluation.

**Table 15:** Bowel preparation adherence outcomes.

Outcome	Combined group (*n* = 131)	Control group (*n* = 126)	Statistic	*P* value
Bowel preparation completed within the prescribed time window, *n* (%)	121 (92.37)	106 (84.13)	χ^2^ = 4.247	0.039
Compliance with low-residue diet, *n* (%)	122 (93.13)	113 (89.68)	χ^2^ = 0.965	0.326
Reported difficulty completing the regimen, *n* (%)	23 (17.56)	41 (32.54)	χ^2^ = 7.738	0.005
Need for rescue preparation measures, *n* (%)	2 (1.53)	4 (3.17)	Fisher exact	0.431

Data are presented as *n* (%). Categorical variables were compared using the chi-square test or Fisher exact test.

**Table 16:** Supplementary safety outcomes.

Outcome	Combined group (*n* = 131)	Control group (*n* = 126)	*P* value
Serious related adverse events, *n* (%)	0 (0.00)	0 (0.00)	Not estimable
Preparation-related colonoscopy discontinuation, *n* (%)	0 (0.00)	0 (0.00)	Not estimable
Clinically apparent dehydration, *n* (%)	0 (0.00)	1 (0.79)	0.490
Severe abdominal pain requiring medication, *n* (%)	1 (0.76)	2 (1.59)	0.614
Aspiration events, *n* (%)	0 (0.00)	0 (0.00)	Not estimable
Preparation-related emergency management, *n* (%)	0 (0.00)	1 (0.79)	0.490
Electrolyte disturbance requiring intervention, *n* (%)	0 (0.00)	0 (0.00)	Not estimable

Data are presented as *n* (%). Fisher exact test was used when applicable. This table reflects short-term clinical safety events identifiable from medical records. Paired pre- and post-bowel-preparation data for serum potassium, sodium, creatinine, blood urea nitrogen, and estimated glomerular filtration rate were not completely available; therefore, this table cannot replace a systematic laboratory safety evaluation.

### 3.13 Subgroup analysis of overall gastrointestinal adverse reactions

Subgroup analyses demonstrated consistent directional reduction in overall gastrointestinal adverse reaction risk with the combined regimen across strata stratified by age, sex, BMI, constipation history, diabetes history and prior colonoscopy history, with no statistically significant interaction effects identified. Stratification by age, sex, BMI and constipation history uniformly indicated lower gastrointestinal adverse reaction risk in the combined group; interaction testing yielded all *P* > 0.05 across subgroups (Table 17, Figure 7).

**Table 17:** Subgroup analysis of overall gastrointestinal adverse reactions.

Subgroup	*n*	Adjusted OR	95%CI	*P* for interaction
Overall	257	0.30	0.17–0.56	
Age <60 years	143	0.29	0.14–0.60	0.783
Age ≥60 years	114	0.34	0.15–0.78	
Male	130	0.31	0.14–0.69	0.912
Female	127	0.30	0.13–0.68	
BMI <24 kg/m²	136	0.28	0.13–0.61	0.694
BMI ≥24 kg/m²	121	0.35	0.15–0.79	
No constipation history	218	0.32	0.17–0.62	0.833
Constipation history	39	0.27	0.07–1.02	

OR, odds ratio; CI, confidence interval; GI, gastrointestinal. Adjusted ORs indicate the odds of any gastrointestinal adverse reaction in the combined group relative to the control group.

**Figure 7:**
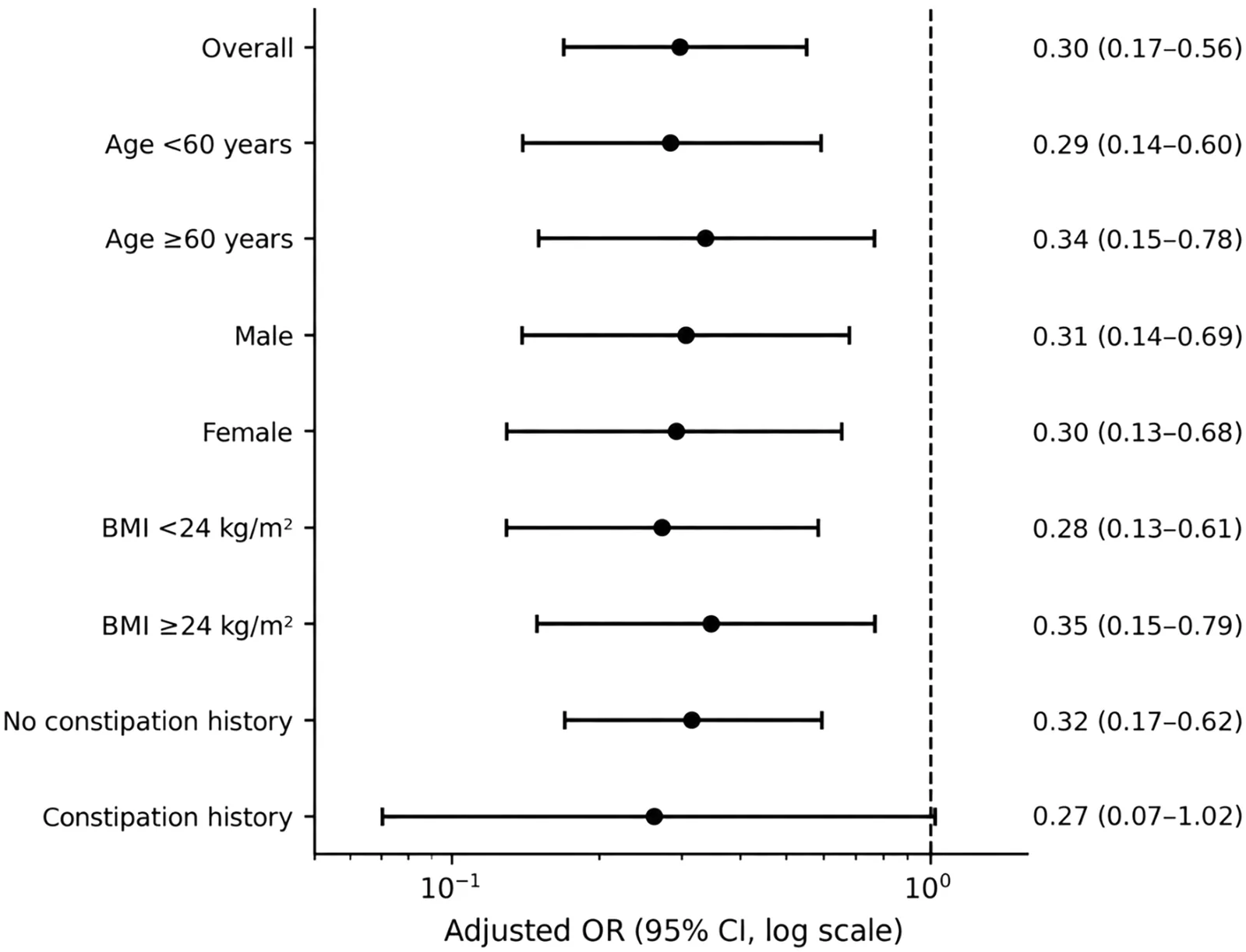
Subgroup analysis of overall gastrointestinal adverse reactions GI, gastrointestinal; OR, odds ratio; CI, confidence interval. Adjusted ORs indicate the odds of any gastrointestinal adverse reaction in the combined group relative to the control group.

## 4 Discussion

Bowel preparation quality constitutes the core evaluation metric for pre-colonoscopy preparation regimens. In recent years, low-volume PEG regimens and combination strategies with adjuvant laxatives have garnered increasing research attention. A randomized controlled trial by Barkun et al. (11) demonstrated that split-dose low-volume PEG regimens achieve non-inferior adequate bowel preparation rates vs. split-dose high-volume PEG, alongside superior patient tolerance and greater regimen reuse willingness. Sirinawasatien et al. (12) compared 2 L split-dose PEG plus lubiprostone against 4 L split-dose PEG, confirming acceptable cleansing efficacy and no incremental adverse events with low-volume PEG combined with secretagogues. A Chinese-population trial by Cheng et al. (13) reported equivalent cleansing efficacy between 3 L and standard 4 L PEG regimens, with improved patient acceptability for the 3 L formulation. Collectively, these studies confirm that total PEG volume is not the sole determinant of bowel preparation quality; medication timing, adjuvant pharmacotherapy, intestinal motility status, patient baseline characteristics, and compliance jointly modulate final cleansing outcomes. In the present study, despite the reduced PEG-ELS volume, rhubarb co-administration may compensate for insufficient cleansing propulsion inherent to low-volume PEG by accelerating defecation and intestinal content evacuation, thereby sustaining overall bowel cleansing quality.

It should be emphasized that low-volume PEG regimens are not universally applicable to all patients. A multicenter randomized trial by Yan et al. (14) identified superior adequate preparation rates and BBPS scores with 3 L split-dose PEG vs. 2 L PEG among individuals with BMI ≥ 24 kg/m^2^, with more pronounced advantages in overweight and constipated patients. This highlights the necessity of individualized risk stratification before low-volume regimen selection, with particular attention to BMI, constipation history, diabetes, prior inadequate bowel preparation, and examination scheduling. Baseline characteristics, including BMI, constipation history, diabetes history, prior abdominal surgery history, prior colonoscopy history, and the interval from preparation to colonoscopy, were balanced between groups in this study. Importantly, patients with constipation were not excluded from the primary cohort. A total of 39 patients with a documented history of constipation were included, comprising 21 patients in the combined group and 18 in the control group. Because rhubarb has stimulant laxative properties, patients with impaired intestinal transit might theoretically derive greater benefit from the combined regimen. In the exploratory constipation subgroup analysis, adequate bowel preparation was achieved in 95.24% (20/21) of patients receiving low-volume PEG-ELS plus rhubarb compared with 83.33% (15/18) receiving conventional PEG-ELS, corresponding to an absolute difference of 11.91 percentage points. Although this difference did not reach statistical significance, the combined regimen significantly shortened both the time to first defecation and total bowel evacuation time. Nevertheless, the constipation subgroup comprised only 39 patients, and constipation subtype was not systematically documented. Therefore, these findings should be regarded as exploratory and cannot establish a specific benefit in patients with slow-transit constipation. Prospective randomized studies specifically enrolling patients with chronic or objectively defined slow-transit constipation are warranted. As this was a retrospective observational study, unmeasured residual confounding cannot be fully eliminated. Future stratified research targeting high-risk populations, including patients with elevated BMI, chronic constipation, diabetic gastrointestinal dysmotility, or recurrent inadequate bowel preparation, is required to delineate the applicable scope of low-volume PEG-ELS plus rhubarb.

The combined group exhibited lower defoaming scores, indicating reduced foam/bubble interference with endoscopic visualization. During colonoscopy, foam, mucus, turbid fluid, and fine fecal residue can impede mucosal visualization, increase intraprocedural irrigation and suction requirements, and potentially obscure flat lesions, diminutive polyps, and right-sided colonic lesions. A systematic review and meta-analysis by Cao et al. (15) validated oral simethicone for improving colonoscopy quality, particularly by mitigating foam-related visual obstruction. A subsequent study by Cao et al. (16) further demonstrated that the interval between simethicone ingestion and colonoscopy, the lag from final preparation to examination, and procedural start time independently modulate bowel preparation quality. These findings establish foam control and luminal visualization clarity as important contributors to colonoscopic diagnostic performance. Distinct from simethicone, which is a direct anti-foaming agent, the lower foam burden observed with rhubarb in this study may be secondary to accelerated intestinal evacuation. Rhubarb contains sennosides, rhein, emodin, and other anthraquinone constituents with well-documented purgative activity through enhanced intestinal peristalsis, consistent with prior pharmacological research (6–8, 17). During PEG-ELS bowel preparation, rhubarb may amplify intestinal propulsive motility and accelerate clearance of luminal contents, mucus, residual fluid, and fine fecal debris, thereby reducing foam and turbid fluid retention and indirectly improving endoscopic visualization. This study did not quantify luminal foam composition, residual fluid volume, irrigation/suction volume, or mucus residue grading, precluding definitive mechanistic elucidation of the observed defoaming benefit. Standardized prospective trials documenting intraprocedural irrigation volume, suction frequency, and residual luminal fluid grading are needed to determine whether rhubarb-mediated accelerated evacuation alleviates foam interference and improves mucosal visualization.

Although bubble-clearance time was significantly shorter in the combined group, this advantage did not translate into a statistically significant reduction in total insertion time. The mean reduction in bubble-clearance time was approximately 8.63 s, accounting for only a small proportion of the overall insertion procedure. Cecal insertion time is additionally influenced by colonic anatomy, looping and angulation, patient cooperation, position changes, abdominal compression, sedation status, previous abdominal surgery, and endoscopist technique. Therefore, the improved defoaming performance observed in the combined group primarily reflects reduced time required for bubble management and clearer mucosal visualization rather than a measurable improvement in overall insertion efficiency.

The markedly shorter time to first defecation and total bowel evacuation time in the combined group indicate that low-volume PEG-ELS plus rhubarb accelerates the bowel preparation response. Rhubarb anthraquinone constituents, traditionally used for purgation, may stimulate intestinal motility, modulate luminal water transport, and alter intestinal microecology to enhance laxative effects. Gao et al. (17) demonstrated rhubarb-mediated mitigation of constipation-associated intestinal inflammation, short-chain fatty acid metabolic dysregulation, and gut microbiota imbalance. Tajika et al. (18) identified significant variation in patient acceptability across different low-volume PEG split-dose administration schedules. However, an important distinction exists between chronic constipation management and short-term pre-colonoscopy bowel preparation. The present study cannot establish causal mechanistic pathways linking rhubarb to accelerated defecation, and targeted preclinical and clinical mechanistic investigations are warranted to validate these preliminary observational findings.

Gastrointestinal tolerance represents a decisive factor in the completion of bowel preparation and willingness to undergo repeat colonoscopy. Although high-volume PEG provides stable cleansing efficacy, large fluid intake frequently triggers nausea, vomiting, abdominal distension, and medication non-adherence. A systematic review and meta-analysis by Ma and Fang (19) reported comparable cleansing efficacy between high- and low-volume PEG combination regimens, with superior regimen reuse willingness and reduced nausea, vomiting, and abdominal pain in low-volume cohorts. A real-world study by Occhipinti et al. (20) similarly validated the feasibility of low-volume PEG plus bisacodyl for balancing cleansing efficacy and tolerance. Consistent with these published data, the present study identified lower incidences of nausea, vomiting, abdominal distension, and overall gastrointestinal adverse reactions in the combined group, with no intergroup difference in abdominal pain. This finding may reflect the reduced total PEG-ELS fluid volume together with rhubarb-mediated acceleration of intestinal content clearance, which may minimize luminal stasis and distension. However, rhubarb itself carries theoretical risks of abdominal cramping, severe diarrhea, and water-electrolyte disturbance. Importantly, the absence of clinically documented severe adverse events should not be interpreted as evidence of complete biochemical safety. Because this was a retrospective study, paired laboratory testing before and after bowel preparation was not routinely performed, and complete data for serum sodium, potassium, creatinine, blood urea nitrogen, and estimated glomerular filtration rate were unavailable. Therefore, mild or subclinical electrolyte disturbances or transient renal function changes may have been missed. Future prospective studies should incorporate standardized pre- and post-preparation measurements of serum electrolytes and renal function, particularly in elderly patients and those with renal impairment, cardiovascular disease, or a high risk of fluid-electrolyte disturbance, to more comprehensively establish the safety profile of this regimen. Clinical use should therefore remain cautious in elderly patients, individuals with renal or cardiac insufficiency, those at high risk of electrolyte disturbance, and chronic users of anthraquinone laxatives.

Patient-reported outcomes constitute a key supplementary endpoint in this investigation, with significantly higher satisfaction and regimen reuse willingness documented in the combined group. Contemporary bowel preparation research increasingly prioritizes patient-reported metrics. Tontini et al. (21) affirmed patient-reported outcome measures as integral to regimen evaluation through assessment of taste, ease of administration, acceptability, and repeat-use intention. Serradesanferm et al. (22) incorporated adenoma detection rate, tolerance, safety, and satisfaction as composite endpoints in screening-population trials of ultra-low-volume bowel preparation. Shaukat et al. (23) emphasized bowel preparation quality, withdrawal time, and endoscopic technique as core modulators of adenoma detection performance. Accordingly, regimen value cannot be quantified exclusively by BBPS scores; comprehensive evaluation should integrate treatment completion feasibility, gastrointestinal discomfort severity, patient satisfaction, and willingness to repeat the preparation. For patients requiring screening, surveillance, or long-term colonoscopic follow-up, an improved preparation experience may enhance colonoscopy uptake and longitudinal adherence to surveillance protocols.

This study additionally analyzed adenoma, advanced adenoma, and right-sided colon adenoma detection rates, with no statistically significant intergroup differences observed across all lesion endpoints. These findings indicate that low-volume PEG-ELS plus rhubarb reduced the fluid-intake burden and improved tolerance without an observed reduction in pathological lesion detection rates. Adenoma detection rate serves as a benchmark metric for colonoscopy quality assurance, with right-sided lesions particularly vulnerable to impairment secondary to suboptimal bowel preparation, foam residue, poor mucosal exposure, and inadequate withdrawal inspection. Zessner-Spitzenberg et al. (24) linked bowel preparation quality to colorectal cancer identification and disease-specific mortality within a national screening program. Li et al. (25) demonstrated that excellent bowel preparation confers no incremental adenoma/polyp detection benefit relative to adequate preparation, whereas inadequate preparation substantially impairs lesion identification. Park and Cha (26) summarized adequate bowel preparation rate, adenoma detection rate, withdrawal time, and cecal intubation rate as foundational colonoscopy quality indicators. The uniformly high adequate preparation rates in both cohorts in the present study likely explain the absence of intergroup differences in polyp, adenoma, advanced adenoma, and right-sided adenoma detection.

Supplementary analyses of compliance and severe safety events revealed higher rates of on-schedule preparation completion and lower self-reported regimen difficulty in the combined group. No severe bowel preparation-related adverse events, premature colonoscopy termination secondary to preparation failure, aspiration, or electrolyte disturbances requiring medical intervention were recorded in either cohort. Spada et al. (27) proposed comprehensive evaluation of novel or low-volume bowel preparation regimens by integrating cleansing efficacy, compliance, patient acceptability, and safety. Hong and Baek (28) advocated balanced assessment of cleansing performance, tolerance, and special-population risk stratification during regimen optimization. Collectively, these findings support the clinical feasibility and acceptable short-term tolerability of low-volume PEG-ELS plus rhubarb in the present cohort. However, retrospective reliance on medical and nursing records may have resulted in underreporting of mild or unprompted adverse symptoms. In addition, systematic paired electrolyte, renal function, and hydration-related laboratory testing before and after bowel preparation was not performed, precluding rigorous evaluation of regimen-specific effects on water-electrolyte homeostasis.

This research has several clinical implications. The study population reflects routine real-world colonoscopy practice, supporting the relevance of the findings to everyday clinical care. Beyond conventional BBPS scoring and adequate preparation rates, the integrated assessment of defoaming performance, defecation kinetics, gastrointestinal adverse reactions, compliance, short-term safety, satisfaction, and willingness to reuse the regimen provides a broader evaluation of bowel preparation performance. Clinically, low-volume PEG-ELS plus rhubarb may represent an individualized candidate regimen for appropriately selected patients, particularly those with documented intolerance to high-volume PEG, previous difficulty completing bowel preparation, or low willingness to undergo repeat colonoscopy. Nevertheless, individualized risk stratification remains necessary for patients with elevated BMI, chronic constipation, diabetic gastrointestinal dysmotility, recurrent inadequate bowel preparation, advanced age with multimorbidity, or a high risk of water-electrolyte disturbance. Low-volume regimens should not universally replace conventional high-volume PEG without appropriate clinical risk assessment.

This study has several limitations. First, complete paired pre- and post-preparation electrolyte and renal function data were unavailable because routine measurement of serum sodium, potassium, creatinine, blood urea nitrogen, and estimated glomerular filtration rate within 24–72 h after bowel preparation was not standard practice at our institution. Consequently, the safety assessment was primarily limited to clinically apparent short-term adverse events documented in medical and nursing records. Mild, asymptomatic, or transient electrolyte disturbances, dehydration, or renal function changes may therefore have been underdetected. Accordingly, the absence of documented serious adverse events should not be interpreted as evidence of complete biochemical safety. Future prospective studies should incorporate standardized laboratory monitoring of serum electrolytes, renal function, and hydration status before and after bowel preparation, particularly in elderly patients and individuals with renal impairment, cardiovascular disease, or a high risk of fluid-electrolyte disturbance. Second, as a single-center retrospective cohort study, bowel preparation regimens were not randomly assigned, and treatment selection may therefore have been influenced by physician preference, anticipated patient tolerance, expected adherence, previous bowel preparation experience, examination scheduling, anesthesia modality, and underlying comorbidities. This introduces the possibility of selection bias and confounding by indication. For example, clinicians may have been more likely to choose the low-volume regimen for patients perceived to be healthier, more tolerant of the preparation process, or more likely to comply with instructions, which could have contributed to the observed differences in gastrointestinal tolerance and patient-reported outcomes. Although multivariable adjustment, propensity score matching, and inverse probability of treatment weighting were applied and achieved satisfactory balance in measured baseline characteristics, these methods can address only measured confounders and cannot eliminate bias from unmeasured or incompletely documented factors. Therefore, residual confounding cannot be excluded, and the observed associations should not be interpreted as definitive causal effects. Future adequately powered prospective multicenter randomized controlled trials are needed to confirm the comparative efficacy, tolerance, and safety of low-volume PEG-ELS plus rhubarb. Third, the sample size was relatively limited, particularly for subgroup analyses. Only 39 patients had a documented history of constipation, and the exploratory constipation subgroup analysis was not powered to establish superiority or a differential treatment effect. In addition, constipation phenotype was not systematically documented in the retrospective records; therefore, patients with slow-transit constipation could not be specifically identified. Although the combined regimen showed favorable bowel evacuation characteristics in patients with constipation, these findings should be regarded as exploratory and require confirmation in prospective studies specifically enrolling patients with chronic or objectively defined slow-transit constipation. Fourth, several outcomes were derived retrospectively from routine clinical, endoscopic, nursing, and follow-up records. Although predefined definitions, dual data extraction, and cross-verification were used to reduce information bias, incomplete documentation of mild gastrointestinal symptoms, patient-reported experience, or procedural details remains possible. Procedural factors such as irrigation volume and suction frequency were not systematically captured in the original records; therefore, the relationship between improved defoaming and overall colonoscopy efficiency cannot be fully elucidated. Fifth, although polyp, adenoma, advanced adenoma, and right-sided adenoma detection rates were evaluated, pathological outcomes were abstracted from routine clinical pathology reports without centralized blinded pathological review. More detailed lesion-level analyses, including sessile serrated lesions, flat adenomas, lesion size categories, and adenoma burden per patient, were not performed. In addition, the relatively small number of advanced adenoma events limited statistical power for analyses of high-risk lesion detection. Finally, the single-center design and restricted sample size limit the generalizability of the findings, particularly to elderly patients, those with severe or slow-transit constipation, chronic kidney disease, cardiac insufficiency, recurrent inadequate bowel preparation, and individuals at high risk of fluid-electrolyte disturbance. Larger prospective multicenter randomized controlled trials with standardized laboratory safety monitoring, detailed procedural measurements, and prespecified analyses of high-risk patient subgroups are warranted to confirm the cleansing efficacy, gastrointestinal tolerance, patient acceptability, lesion detection performance, and biochemical safety of low-volume PEG-ELS plus rhubarb.

In summary, low-volume PEG-ELS with rhubarb, when compared with standard 3-L PEG-ELS, provided similar bowel cleansing quality and lesion detection capability while offering better gastrointestinal tolerability, quicker defecation onset, less foam interference, and higher patient satisfaction and willingness to reuse the same preparation. However, owing to the retrospective, single-center design and the lack of comprehensive laboratory safety data, these findings should be regarded as hypothesis-generating and require confirmation in prospective, multicenter randomized controlled trials.

## Data Availability

The raw data supporting the conclusions of this article will be made available by the authors, without undue reservation.

## References

[1] Jacobson BC Anderson JC Burke CA Dominitz JA Gross SA May FP. Optimizing bowel preparation quality for colonoscopy: consensus recommendations by the US multi-society task force on colorectal cancer. Am J Gastroenterol. (2025) 120(4):738–64. 10.14309/ajg.0000000000003287 40035345

[2] Wang L Zhang Y Li J Ran Y Wang X Ma X. Efficacy of polyethylene glycol electrolyte powder combined with linaclotide for colon cleansing in patients with chronic constipation undergoing colonoscopy: a multicenter, single-blinded, randomized controlled trial. Clin Transl Gastroenterol. (2024) 15(6):e1. 10.14309/ctg.0000000000000708 PMC11196075 38713137

[3] Liu WQ Shu L Zhou X Wang XF Liu S Shi ZH. Evaluation of the efficacy of polyethylene glycol in combination with different doses of linaclotide in a fractionated bowel preparation for colonoscopy: a prospective randomized controlled study. Int J Colorectal Dis. (2024) 39(1):143. 10.1007/s00384-024-04718-4 39289199 PMC11408392

[4] Zhang X Chen Y Chen Y Zhu W Tang C Lamm S. Polyethylene glycol combined with lactulose has better efficacy than polyethylene glycol alone in bowel preparation before colonoscopy: a meta-analysis. Clinics (Sao Paulo). (2023) 78:100172. 10.1016/j.clinsp.2023.100172 37019039 PMC10126707

[5] Tangvoraphonkchai K Manasirisuk W Sawadpanich K Suttichaimongkol T Mairiang P. Lubiprostone plus polyethylene glycol electrolyte lavage solution (PEG-ELS) versus PEG-ELS for bowel preparation in chronic constipation: a randomized controlled trial. Sci Rep. (2023) 13(1):16265. 10.1038/s41598-023-43598-6 37759079 PMC10533515

[6] Zhang R Huang C Wu F Fang K Jiang S Zhao Y. Review on melanosis coli and anthraquinone-containing traditional Chinese herbs that cause melanosis coli. Front Pharmacol. (2023) 14:1160480. 10.3389/fphar.2023.1160480 37214441 PMC10193150

[7] Wen Y Yan PJ Fan PX Lu SS Li MY Fu XY. The application of rhubarb concoctions in traditional Chinese medicine and its compounds, processing methods, pharmacology, toxicology and clinical research. Front Pharmacol. (2024) 15:1442297. 10.3389/fphar.2024.1442297 39170703 PMC11335691

[8] Neyrinck AM Rodriguez J Taminiau B Herpin F Cani PD Daube G. Constipation mitigation by rhubarb extract in middle-aged adults is linked to gut microbiome modulation: a double-blind randomized placebo-controlled trial. Int J Mol Sci. (2022) 23(23):14685. 10.3390/ijms232314685 36499011 PMC9738964

[9] Lai EJ Calderwood AH Doros G Fix OK Jacobson BC. The Boston bowel preparation scale: a valid and reliable instrument for colonoscopy-oriented research. Gastrointest Endosc. (2009) 69(3 Pt 2):620–5. 10.1016/j.gie.2008.05.057 19136102 PMC2763922

[10] McNally PR Maydonovitch CL Wong RK. The effectiveness of simethicone in improving visibility during colonoscopy: a double-blind randomized study. Gastrointest Endosc. (1988) 34(3):255–8. 10.1016/s0016-5107(88)71324-3 3292345

[11] Barkun AN Martel M Epstein IL Hallé P Hilsden RJ James PD. The bowel CLEANsing national initiative: high-volume split-dose vs low-volume split-dose polyethylene glycol preparations: a randomized controlled trial. Clin Gastroenterol Hepatol. (2022) 20(6):e1469–77. 10.1016/j.cgh.2021.09.005 34509641

[12] Sirinawasatien A Sakulthongthawin P Chanpiwat K Chantarojanasiri T. Bowel preparation using 2-L split-dose polyethylene glycol regimen plus lubiprostone versus 4-L split-dose polyethylene glycol regimen: a randomized controlled trial. BMC Gastroenterol. (2022) 22(1):424. 10.1186/s12876-022-02497-2 36115968 PMC9482745

[13] Cheng P Chen Q Li J Pang L Feng C Wang N. 3 Liters of polyethylene glycol vs. Standard bowel preparation have equal efficacy in a Chinese population: a randomized, controlled trial. Am J Transl Res. (2022) 14(8):5641–50. 36105041 PMC9452343

[14] Yan H Huang H Yang D Chen Z Liu C Huang Z. 3 L split-dose polyethylene glycol is superior to 2 L polyethylene glycol in colonoscopic bowel preparation in relatively high-BMI (≥24 kg/m2) individuals: a multicenter randomized controlled trial. BMC Gastroenterol. (2023) 23(1):427. 10.1186/s12876-023-03068-9 38053082 PMC10698874

[15] Cao RR Wang L Gao C Pan JH Yoshida EM Li HY. Effect of oral simethicone on the quality of colonoscopy: a systematic review and meta-analysis of randomized controlled trials. J Dig Dis. (2022) 23(3):134–48. 10.1111/1751-2980.13084 35075814

[16] Cao R Meng F Li C Chen H Gao C Li H. Shortening oral simethicone-to-colonoscopy interval increases bowel preparation quality. Med Sci Monit. (2024) 30:e943972. 10.12659/MSM.943972 38907516 PMC11305093

[17] Gao H He C Hua R Liang C Wang B Du Y. Underlying beneficial effects of rhubarb on constipation-induced inflammation, disorder of gut microbiome and metabolism. Front Pharmacol. (2022) 13:1048134. 10.3389/fphar.2022.1048134 36545319 PMC9760883

[18] Tajika M Tanaka T Ishihara M Hirayama Y Onishi S Mizuno N. Split-dose low-volume polyethylene glycol is non-inferior but less preferred compared with same-day bowel preparation for afternoon colonoscopy. Nagoya J Med Sci. (2021) 83(4):787–99. 10.18999/nagjms.83.4.787 34916722 PMC8648521

[19] Ma G Fang X. The safety and effects of high- and low-volume polyethylene glycol bowel preparation methods before colonoscopy on bowel cleanliness: a systematic review and meta-analysis. J Gastrointest Oncol. (2023) 14(4):1759–69. 10.21037/jgo-23-581 37720457 PMC10502556

[20] Occhipinti V Soriani P Bagolini F Milani V Rondonotti E Annunziata ML. Efficacy and tolerability of high and low-volume bowel preparation compared: a real-life single-blinded large-population study. World J Gastrointest Endosc. (2021) 13(12):659–72. 10.4253/wjge.v13.i12.659 35070027 PMC8716982

[21] Tontini GE Spada C Uebel P Cannizzaro R Ciprandi G Vecchi M. Assessment of patient-reported outcome measures in patients undergoing bowel preparation with mannitol for colonoscopy: the SATISFACTION study. JGH Open. (2025) 9(8):e70237. 10.1002/jgh3.70237 40786127 PMC12332180

[22] Serradesanferm A Torá-Rocamora I Pozo À Ocaña T Diaz M Moreira R. Adenoma detection rate and tolerability of 2 ultra-low-volume bowel preparations in screening: a noninferiority randomized controlled trial. Gastrointest Endosc. (2025) 101(1):158–167.e7. 10.1016/j.gie.2024.07.007 39069266

[23] Shaukat A Tuskey A Rao VL Dominitz JA Murad MH Keswani RN. Interventions to improve adenoma detection rates for colonoscopy. Gastrointest Endosc. (2022) 96(2):171–83. 10.1016/j.gie.2022.03.026 35680469

[24] Zessner-Spitzenberg J Waldmann E Rockenbauer LM Klinger A Klenske E Penz D. Impact of bowel preparation quality on colonoscopy findings and colorectal cancer deaths in a nation-wide colorectal cancer screening program. Am J Gastroenterol. (2024) 119(10):2036–44. 10.14309/ajg.0000000000002880 39007693 PMC11446535

[25] Li Y Meng F Cao R Gao C Su D Wang K. Excellent bowel preparation quality is not superior to good bowel preparation quality for improving adenoma/polyp detection rate. Clin Med Insights Oncol. (2024) 18:11795549241229190. 10.1177/11795549241229190 38332773 PMC10851725

[26] Park SB Cha JM. Quality indicators in colonoscopy: the chasm between ideal and reality. Clin Endosc. (2022) 55(3):332–8. 10.5946/ce.2022.037 35656625 PMC9178135

[27] Spada C Fiori G Uebel P Tontini GE Cesaro P Grazioli LM. Oral mannitol for bowel preparation: a dose-finding phase II study. Eur J Clin Pharmacol. (2022) 78(12):1991–2002. 10.1007/s00228-022-03405-z 36287232 PMC9649514

[28] Hong SM Baek DH. Updated bowel preparation regimens for colonoscopy: benefits and drawbacks. Clin Endosc. (2026) 59(3):345–55. 10.5946/ce.2025.201 41409028 PMC13254635

